# Primary thyroid lymphoma: A single-center experience

**DOI:** 10.3389/fendo.2023.1064050

**Published:** 2023-02-09

**Authors:** Jin Seok Lee, Su-Jin Shin, Hyeok Jun Yun, Seok Mo Kim, Hojin Chang, Yong Sang Lee, Hang-Seok Chang

**Affiliations:** ^1^Department of Surgery, Thyroid Cancer Center, Gangnam Severance Hospital, Institute of Refractory Thyroid Cancer, Yonsei University College of Medicine, Seoul, Republic of Korea; ^2^Department of Pathology, Gangnam Severance Hospital, Yonsei University College of Medicine, Seoul, Republic of Korea

**Keywords:** thyroid lymphoma, Hodgkin’s lymphoma, non-Hogdkin’s lymphoma, Hashimoto’s thyroiditis, fine needle aspirate

## Abstract

**Background:**

Primary thyroid lymphoma (PTL) is a very rare entity accounting for 5% of all thyroid malignancies and less than 2% of lymphomas. PTLs are classified as non-Hodgkin’s B-cell lymphomas in the majority of cases, although Hodgkin’s lymphoma of the thyroid has also been identified. This study aimed to identify the clinical, biochemical, and pathological features of primary thyroid lymphomas.

**Methods:**

From January 2008 to December 2020, data from patients diagnosed with PTL treated at the Gangnam Severance Hospital, including clinical, biochemical, and pathological features of thyroid lymphomas, were assessed.

**Results:**

Of 10 patients, nine women and one man, with a median age of 62 (range, 44–82) years were included. Fine needle aspiration biopsy was performed in nine patients and surgical resection was performed in one patient without biopsy. Excisional and surgical biopsies were performed in all patients, including five who underwent excisional biopsy and five who underwent thyroidectomy. Histological analyses revealed that all 10 lymphomas were non-Hodgkin B-cell lymphoma; six patients had diffuse large B-cell lymphoma, three had mucosa-associated lymphoid tissue lymphoma, and one had Burkitt lymphoma. Four patients received chemotherapy, two were treated with chemoradiation therapy, one received radiation therapy only, one did not require more treatment after surgery, one refused treatment, and one was transferred to another hospital.

**Conclusions:**

Although PTLs are scarce, clinicians should be aware of this rare entity and evaluate and treat PTLs on an individual basis.

## Introduction

Primary thyroid lymphoma (PTL) is an extremely rare malignancy of the thyroid gland, accounting for 5% of thyroid malignancies and 2% of extranodal lymphomas, with an estimated annual incidence of 2 per 1 million (1). Thyroid lymphoma usually manifests as a rapidly growing mass in the neck, causing compression symptoms (2). Hashimoto’s thyroiditis is known to increase the relative risk of developing thyroid lymphoma; nevertheless, only 0.5% of all Hashimoto’s thyroiditis cases develop PTL (3, 4). Because of this underlying risk factor, PTL typically develops more frequently in women than in men (8:1) (5) and usually in the sixth or seventh decade of life (6). Thyroid lymphoma originates from lymphocytes, rather than the follicular cells of thyroid, most commonly the B lymphocytes (7). For this reason, usually, fine needle aspiration (FNA) cytology may be sufficient to diagnose most thyroid masses; however, its accuracy in thyroid lymphoma has been found to be quite variable (2, 8, 9) because of the large sample size needed for immunohistochemistry, for diagnosis with subtype confirmation (10). It is important to make a quick and accurate diagnosis by cytology for an overall prognosis and treatment plan, which will depend on the lymphoma subtype. Core needle biopsy is strongly considered in doubtful cases for accurate diagnosis with immunohistochemistry, which is used as the method of choice as a reliable diagnostic tool (11, 12). With the rapid enlarging neck mass with compression, PTL and anaplastic thyroid cancer are difficult for the clinician to distinguish between the two based on history (13). Even in ultrasound reporting, PTL and anaplastic thyroid cancer show significant overlap with solid mass that is hypoechoic (14). Earlier, PTLs was compared to aggressive types of thyroid malignancies such as anaplastic thyroid cancer in overall survival rates. However, with recent advances in treatment, PTLs have been shown to be highly responsive to therapeutic interventions and have excellent overall survival rates (15, 16).

This study aimed to introduce and identify the clinical, biochemical, and pathological features of PTLs treated at a single institution. This information can aid clinicians to more accurately determine the pretest probability of thyroid lymphoma and select the diagnostic tests with the highest yield for obtaining a final diagnosis.

## Materials and methods

Patients who were diagnosed with PTL had a pathological diagnosis and underwent surgical intervention, excisional biopsy, or thyroidectomy for precise diagnosis at Gangnam Severance Hospital, Yonsei University College of Medicine, Korea, between January 2008 and December 2020. Medical information, including demographic, clinical, and laboratory data, were extracted from electronic medical records and reviewed retrospectively. Pathologic data were independently reviewed by an expert pathologist using a specimen slide. The laboratory data collected included sensitive thyrotropin (TSH), free thyroxine, serum thyroid peroxidase (TPO) antibody, and thyroglobulin antibody with reference ranges of 0.8–4.7 mIU/L, 0.8–1.7 ng/dL, 5–13.6 IU/mL, and 10–124 IU/mL, respectively. Preoperative ultrasound was performed on all patients. On reports of ultrasound imaging, hypoechoic was considered as having decreased echogenicity relative to adjacent thyroid tissue, and very hypoechoic as decreased echogenicity relative to adjacent musculature. FNA biopsy was defined as a biopsy performed using a needle gauge of 25G under ultrasound–guided. Local anesthesia was not routinely used to prevent pain during FNA, but might be helpful for multiple needle punctures. FNA was performed with the needle oriented either parallel or perpendicular to the US probe. Tissue samples were collected with 6 to 7 “to-and fro” needle movements over 5–10 seconds, with 2–3 mL suction applied. The number of needle passes ranged from 2 to 3. Core needle biopsy was defined as a biopsy performed with a spring-loaded tru-cut configured biopsy needle with a gauge of 18G. All patients underwent core needle or surgical biopsy for an accurate diagnosis with immunohistochemistry which included CD3, CD20, cyclinD1, BCL2, CD10, BCL6, MUM1, Myc, Ki-67, CD5, and EBV *in situ* hybridization.

Hypothyroidism was considered as either an elevated TSH value or levothyroxine replacement therapy at the time of diagnosis. Hashimoto’s thyroiditis was defined as a diagnosis in the medical history. PTL was defined as a disease confined to the neck.

In PTL treatment, the purpose of surgery is mainly for biopsies; excision biopsy and thyroidectomy are typically performed. Chemotherapy usually includes multiple courses of CHOP (cyclophosphamide 750 mg/m^2^, adriamycin 50 mg/m^2^, vincristine 1.4 mg/m^2^, and prednisolone 100 mg/day) with rituximab (375 mg/m^2^). In current study, one patient diagnosed with Burkitt’s lymphoma received R-Hyper CVAD (rituximab 375 mg/m^2^, cyclophosphamide 600 mg/m^2^ daily, vincristine 2 mg/m^2^/day, adriamycin 50 mg/m^2^/day, dexamethasone 40 mg/day, and methotrexate 1 g/m^2^/day). Radiation therapy encompassed the entire neck and the upper mediastinum.

This study was approved by the Institutional Review Board (IRB) of Gangnam Severance Hospital, Yonsei University College of Medicine (IRB protocol: 3-2022-0237). The study protocol was conducted in accordance with the Declaration of Helsinki. Due to the retrospective nature of the study, neither patient approval nor informed consent was required.

## Results

### Clinical, radiologic and diagnostic features

**Figure 1:**
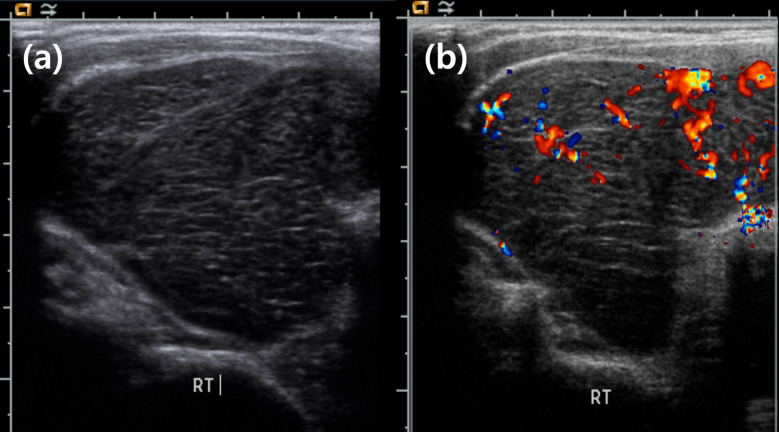
Ultrasound appearance of thyroid lymphoma. **(A)** Large solid mass with very hypoechoic feature. **(B)** Increased vascularity in mass.

The 10 patients comprised nine women and one man with a median age of 62 years, and seven patients were over 60 (range, 44–82) years. All 10 patients performed preoperative ultrasound and seven patients showed solid mass with very hypoechoic texture (**Figure 1**). Three patients had solid mass with hypoechoic echotexture. The typical appearance consisted of increased vascularity relative to normal thyroid parenchyma, no internal calcifications, and variable edge characteristics (from well-defined to ill-defined) (10). FNA biopsy was performed in nine patients, and surgical resection was performed in one patient without biopsy. Cytological results were suspicious for lymphoma for four patients, benign with Hashimoto’s thyroiditis for three patients, benign with intrathyroidal lymph nodes for one patient, and atypia of undetermined significance with the possibility of a lymphoproliferative lesion for one patient. None of the patients who were diagnosed with Hashimoto’s thyroiditis by FNA biopsy had a previous history of Hashimoto’s thyroiditis; one of these two patients had significant TPO antibody elevation (>20.0 IU/mL). Among the 10 patients, one had an elevated TSH level (5.94 mIU/L) at the time of diagnosis for thyroid lymphoma and subclinical hypothyroidism; four patients had the normal range of TSH level and five patients had under the normal range of TSH level. With the value of free T4, four patients had subclinical hyperthyroidism, and one patient had hypothyroidism with levothyroxine replacement therapy at the time of diagnosis (**Table 1**).

**Table 1:** Patient demographics, clinicopathologic features, and initial diagnosis.

Case ID	Sex (F: female, M: male)	Age (years)	TSH (mIU/L)	Free T4 (ng/dL)	TPO Ab (IU/mL)	Tg Ab (IU/mL)	History of Hashimoto’s thyroiditis	Fine needle aspiration
1	F	55	0.1	Subclinical hyperthyroidism (1.8)	–	+	–	Suspicious for lymphoma
2	F	65	5.94	Subclinical hypothyroidism (0.9)	+	–	–	Benign; Hashimoto’s thyroiditis
3	F	64	0.31	Hypothyroidism* (1.4)	+	+	+	No
4	F	69	1.48	Euthyroid (1.6)	–	+	–	Suspicious for lymphoma
5	F	44	1.83	Euthyroid (1.2)	–	+	–	Suspicious for lymphoma
6	F	67	0.7	Subclinical hyperthyroidism (1.3)	–	+	–	Suspicious for lymphoma
7	F	82	0.3	Subclinical hyperthyroidism (1.5)	No	No	–	AUS; possibility of a lymphoproliferative lesion
8	F	68	1.09	Euthyroid (1.1)	–	+	–	Benign; Hashimoto’s thyroiditis
9	M	46	2.48	Euthyroid (0.9)	No	No	–	Benign: Intrathyroidal lymph node
10	F	62	0.06	Subclinical hyperthyroidism (1.9)	–	–	–	Benign; Hashimoto’s thyroiditis

*Patients who took oral levothyroxine were defined as having hypothyroidism. Normal ranges of TSH, 0.86–4.69 mIU/mL; Free T4, 0.8–1.7 ng/dL; TPOAb, 5–13.6 IU/mL; TgAb, 10–124.2 IU/mL. TSH, thyroid-stimulating hormone; Free T4, free thyroxine; TPOAb, thyroid peroxidase antibody; TgAb, thyroglobulin antibody; AUS, atypia of undetermined significance.

### Histological analysis and treatment

**Figure 2:**
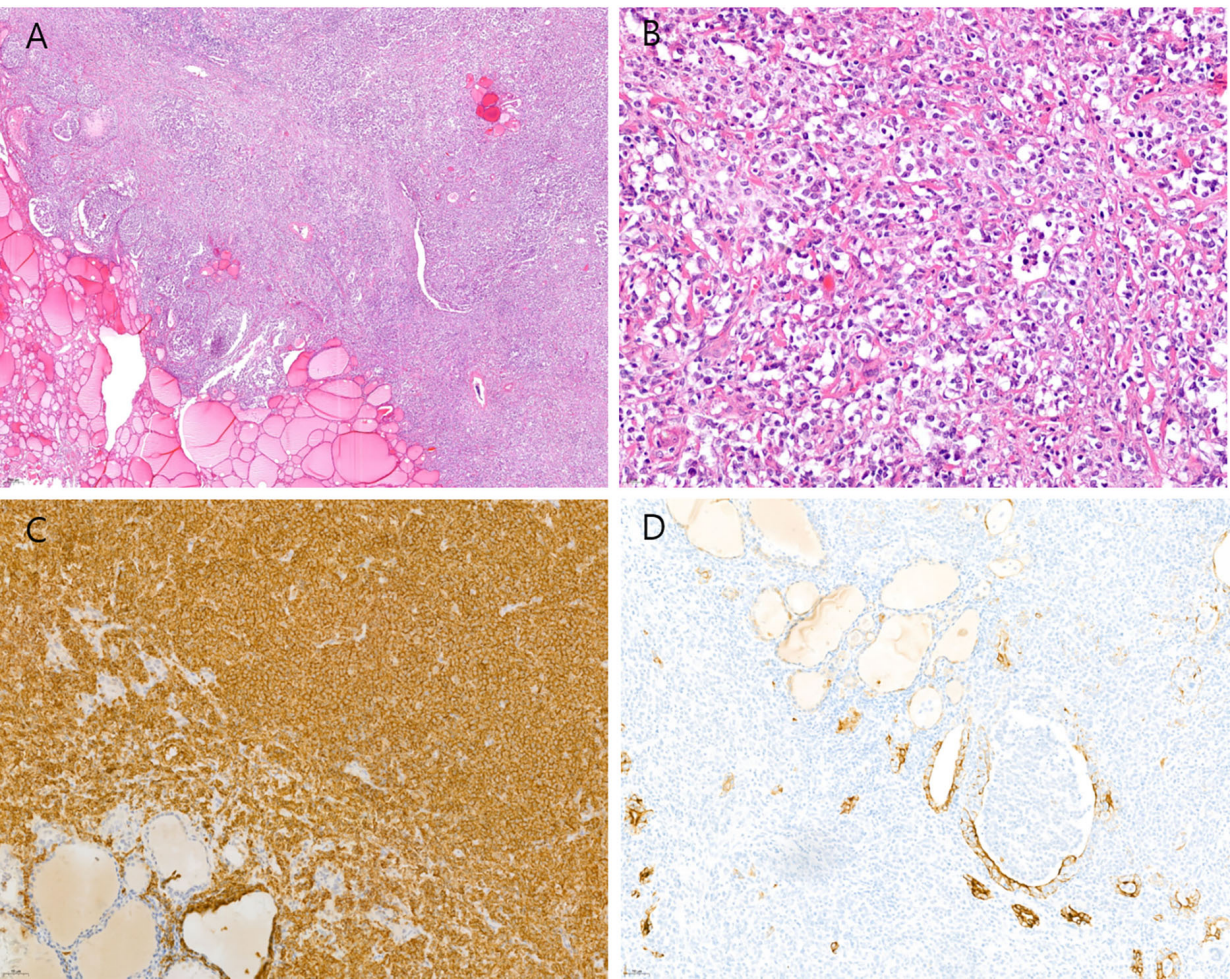
Histological features of diffuse large B cell lymphoma of the thyroid. **(A)** The thyroid gland is diffusely infiltrated by lymphoid cells (hematoxylin-eosin stain, original magnification ×40). **(B)** The large lymphoma cells show vesicular nuclei with distinct nucleoli (hematoxylin-eosin stain, original magnification ×400). **(C)** The tumor cells show strong and diffuse positive immunostaining for CD20 (original magnification ×200). **(D)** The tumor cells have infiltrated into the thyroid follicular epithelium and lumen, confirmed by cytokeratin immunostaining (original magnification ×200).

Excisional and surgical biopsies were performed in all patients, including five patients who underwent excisional biopsy and five patients who underwent thyroidectomy. Histological analyses revealed that all 10 lymphomas were non-Hodgkin B-cell lymphoma, including six patients with diffuse large B-cell lymphoma which thyroid gland was diffusely infiltrated by lymphoid cells and the large lymphoma cells showed vesicular nuclei with distinct nucleoli with strong, diffuse positive immunostaining for CD20 (**Figure 2**). Three patients were mucosa-associated lymphoid tissue (MALT) lymphoma, and one patient was Burkitt lymphoma.

Of all the patients, seven underwent post-surgery treatment, four received chemotherapy, two received chemoradiation therapy, and one received radiation therapy only. The patient who refused any treatment was lost to follow-up, and one patient was transferred to another hospital for treatment. The tenth patient who underwent complete resection did not pursue post-surgical treatment. Among the seven remaining patients, six responded to treatment (**Table 2**).

**Table 2:** Patients’ histological types and clinical outcomes.

Case ID	Histological analysis type	Final diagnosis	Diagnostic method	Treatment after surgical excision	Response	Status
1	Diffuse large B cell lymphoma	Non-Hodgkin’s lymphoma	Lobectomy	Follow-up loss	–	Follow-up loss
2	Diffuse large B cell lymphoma	Non-Hodgkin’s lymphoma	Total thyroidectomy	R-CHOP	CR	Alive
3	Mucosa-associated lymphoid tissue B cell lymphoma	Non-Hodgkin’s lymphoma	Total thyroidectomy	mBACOP	CR	Alive
4	Diffuse large B cell lymphoma	Non-Hodgkin’s lymphoma	Excisional biopsy	R-CHOP	CR	Alive
5	Burkitt lymphoma	Non-Hodgkin’s lymphoma	Lobectomy	Hyper CVAD	CR	Alive
6	Diffuse large B cell lymphoma	Non-Hodgkin’s lymphoma	Excisional biopsy	R-CHOP	CR	Alive
7	Diffuse large B cell lymphoma	Non-Hodgkin’s lymphoma	Excisional biopsy	Follow-up loss	–	Follow-up loss
8	Diffuse large B cell lymphoma	Non-Hodgkin’s lymphoma	Excisional biopsy	R-CHOP	PD (2^nd^ line R-Benda)	Death
9	Mucosa-associated lymphoid tissue B cell lymphoma	Non-Hodgkin’s lymphoma	Excisional biopsy	Radiation only	CR	Alive
10	Mucosa-associated lymphoid tissue B cell lymphoma	Non-Hodgkin’s lymphoma	Total thyroidectomy	No more treatment	–	Alive

R-CHOP, rituximab, cyclophosphamide, adriamycin, vincristine, prednisolone; mBACOP, modified bleomycin, adriamycin, cyclophosphamide, oncovin-vincristine, and prednisolone; Hyper CVAD, hyperfractionated cyclophosphamide, vincristine, adriamycin, dexamethasone; CR, complete remission; PD, progressive disease.

## Discussion

Thyroid lymphoma occurs in patients with Hashimoto’s thyroiditis and is characterized by a rapidly enlarging mass (10). Previous reports have shown that those with Hashimoto’s thyroiditis have a 67-times higher risk of developing thyroid lymphoma than patients with colloid goiter (3). As many as 90% of the patients with PTL have a positive history of thyroid autoimmunity (4) and generally associated with MALT lymphomas than other subtypes, in agreement with the previously postulated mechanism of a slow transformation to malignant due to chronic antigenic stimulation of marginal cells (17). In the cases presented in this study, one of the 10 patients had a history of Hashimoto’s thyroiditis with MALT tissue B-cell lymphoma, and none had diffuse large B-cell lymphoma.

Most thyroid masses undergo FNA biopsy for diagnosis after imaging. FNA biopsy is a good diagnostic method, with controversial effectiveness. In a previous study for thyroid lymphoma (2), it was observed that, among 83 patients with suspected thyroid lymphoma who underwent FNA, 65 (78.3%) were diagnosed accurately, and 10 (12%) had a diagnosis of borderline cytologic results. Thus, in this study, 90% of the patients with thyroid lymphoma either had a suspected diagnosis or were diagnosed correctly based on FNA biopsy. In the study from Italy (18), accuracy of FNA biopsy diagnosis improved with immunohistochemical staining of the FNA-biopsied specimens. Despite the improved accuracy of the thyroid lymphoma subtype diagnosis on the basis of FNA cytology, almost all the cases in the present study required additional tissue sampling for subtype confirmation. Compared with the efficacy of FNA biopsy, core needle biopsy was found to have increased sensitivity for thyroid lymphoma (8). In a meta-analysis study, thyroid lymphoma was misdiagnosed in one case of core needle biopsy compared to 16 cases of FNA biopsy (5 vs. 55%) (8). As for false negatives in the present study, three samples were in core-needle biopsy and 19 in FNA biopsy (7 vs. 29.2%; *P* = 0.048). Seventeen cases with these inaccurate FNAs were given the reassuring label “benign.” Core needle biopsy has been shown to improve the accuracy of thyroid lymphoma diagnosis to 93% from 82% with FNA alone (19). In the study of Matsuzuka et al. (2), the importance of initial selection of an accurate test for diagnosis of thyroid lymphoma was highlighted, with two patients who had delayed diagnosis due to repeated false-negative FNA results dying of progressive lymphomas. In another study, core needle biopsy was commonly delayed by one week after the first FNA biopsy yielded an incorrect diagnosis (10). In addition, there are no major complications after core and FNA biopsies, with only an increase in local discomfort of neck when large-gauge needle are used (20). Sharma et al. (10) stated core needle biopsy should be considered as one of the first diagnostic tests when thyroid lymphoma is highly suspected. To reduce the burden of repeated biopsy and decrease the need for invasive diagnostic procedures, using core needle biopsy as a first step to obtain more tissue may be advantageous, rather than procedures like open excisional biopsy or lobectomy. Unfortunately, in two cases with core biopsy in the present study, a more invasive procedure such as excisional biopsy was needed to ascertain the final diagnosis. This possibility must be considered and discussed with the patient at the time of the initial evaluation.

Traditionally, surgery and radiation therapy (RT) have been considered standard treatments for PTL. However, with high relapse and low survival rates, and the realization that thyroid lymphomas are responsive to chemotherapy and radiation, surgery now plays a limited role (21, 22). According to studies from the Mayo Clinic, high cure rates and disease-free survival are achieved with thyroidectomy and adjuvant RT in thyroid lymphoma (1). Currently, the role of surgery is to obtain more tissues for histological diagnosis. Cervical-mediastinal RT should be the initial treatment of choice for patients with a good prognosis and when the disease is limited to the thyroid. In a case study of 31 patients with primary thyroid MALT lymphoma, a 5-year survival rate of 90% was observed after RT alone (23). Chemotherapy should be performed together RT for high-grade thyroid lymphomas that show extracapsular extension (24). The R-CHOP regimen that contains rituximab has been demonstrated to be the best combination therapy for disease-free survival in thyroid lymphoma (24). In the present study, four patients received only chemotherapy, whereas two patients were treated with chemoradiation therapy. One patient received radiation therapy alone. Only one patient died due to cancer progression, and the other patients achieved complete remission. In the case of surgical intervention, all 10 patients underwent surgical biopsy after FNA or core needle biopsy, even the patients diagnosed as suspicious for lymphoma. High incidence rates of thyroid follicular cell-derived malignancy in Korea make physicians consider open surgical biopsy for accurate and differential diagnosis. The relatively low procedural cost of surgery in Korea is one of some reasons for open surgical biopsy to diagnose accurate thyroidal disease.

One of the limitations of this study is that it was a retrospective review with inherent biases. A small group analysis was performed, and a control group was not included. Therefore, the test characteristics of the various diagnostic techniques could not be calculated.

In conclusion, although PTLs are rare, clinicians should consider the importance of this potential diagnosis when encountering patients with an enlarged neck mass in the setting of Hashimoto’s thyroiditis. FNA biopsy should be considered as the first step, and core needle biopsy or surgical biopsy should be considered promptly if FNA biopsy fails to determine a final diagnosis.

## Data availability statement

The raw data supporting the conclusions of this article will be made available by the authors, without undue reservation.

## Ethics statement

The studies involving human participants were reviewed and approved by Institutional Review Board (IRB) of Gangnam Severance Hospital, Yonsei University College of Medicine (IRB protocol: 3-2022-0237). Written informed consent for participation was not required for this study in accordance with the national legislation and the institutional requirements.

## Author contributions

Study concept and design, YL; data acquisition, analysis, and interpretation, JL and S-JS; drafting of the manuscript, JL, HY, YL, and H-SC. All authors contributed to the article and approved the submitted version.
